# Longitudinal Changes in Occlusal Outcomes and Case Complexity with Invisalign^®^ First in Early Mixed Dentition

**DOI:** 10.3390/dj14060338

**Published:** 2026-06-02

**Authors:** Teresa Pinho, Martim Novais, Maria dos Prazeres Gonçalves

**Affiliations:** 1 UNIPRO—Oral Pathology and Rehabilitation Research Unit, University Institute of Health Science (IUCS—CESPU), 4585-116 Gandra, Portugal; a32026@alunos.cespu.pt; 2 Unit for Multidisciplinary Research in Biomedicine (UMIB), School of Medicine and Biomedical Sciences (ICBAS), University of Porto, Rua Jorge Viterbo Ferreira 228, 4050-313 Porto, Portugal; 3 Associate Laboratory I4HB, Institute for Health and Bioeconomy, University Institute of Health Sciences (IUCS—CESPU), 4585-116 Gandra, Portugal; mprazeres.goncalves@iucs.cespu.pt; 4 UCIBIO—Applied Molecular Biosciences Unit, Translational Toxicology Research Laboratory, University Institute of Health Sciences (1H-TOXRUN, IUCS—CESPU), 4585-116 Gandra, Portugal

**Keywords:** interceptive orthodontic treatment, Invisalign^®^ First, mixed dentition, clear aligner therapy, PAR index, occlusal outcomes

## Abstract

**Background**: Interceptive orthodontic treatment during mixed dentition aims to reduce malocclusion complexity and improve occlusal development. Although Invisalign^®^ First was introduced for Phase I therapy in growing patients, evidence regarding longitudinal occlusal outcomes and maintenance during dental development remains limited. This study described longitudinal changes in occlusal outcomes and case complexity using standardized indices in moderate-to-severe malocclusions. **Methods**: Records of children in early mixed dentition treated with Invisalign^®^ First between 2019 and 2025 were retrospectively analyzed. The full sample comprised 55 patients with complete digital occlusal records at baseline (T0), post-treatment (T1), and after a minimum 18-month follow-up period (T2), enabling longitudinal assessment using the Peer Assessment Rating (PAR) Index. All patients underwent baseline cephalometric assessment for diagnosis and treatment planning. A nested subsample of 47 patients with complete paired T0-T1 cephalometric records was additionally evaluated using a literature-based mixed dentition complexity framework adapted from Align^®^ clinical recommendations. Nonparametric tests (Wilcoxon and Friedman) were applied (α = 0.05). **Results**: Median PAR scores decreased from 22.0 (IQR 17.0–30.0) at T0 to 6.0 (IQR 1.0–8.0) at T1 and 7.0 (IQR 2.0–10.0) at T2, with no significant difference between T1 and T2 (*p* = 0.546). In the cephalometric subsample, global complexity decreased by 79.49 ± 22.43%, with correction rates exceeding 85% for dentoalveolar objectives and reaching 100% for posterior crossbite and open bite. Skeletal and sagittal molar components showed more limited improvement. **Conclusions**: Interceptive treatment with Invisalign^®^ First showed longitudinal reductions in occlusal complexity and high dentoalveolar correction rates, with maintenance of occlusal outcomes during follow-up as patients transitioned from mixed to permanent dentition. These findings should be interpreted as descriptive longitudinal observations within a non-controlled design.

## 1. Introduction

Malocclusions in growing patients rarely self-correct and may worsen during craniofacial development if left untreated [1,2]. Dentoskeletal discrepancies in the transverse, sagittal, and vertical dimensions, such as arch constriction, crowding, molar sagittal discrepancies (Class II and III), anterior open bite, and developing asymmetries, can increase case complexity over time and, in severe cases, contribute to functional imbalance and skeletal disharmony [3,4].

Interceptive orthodontic treatment is defined as an early and time-limited intervention performed during mixed dentition to correct developing malocclusions, guide craniofacial growth, and reduce the need for more complex treatment in adolescence or adulthood [5]. Early correction of transverse deficiencies, sagittal discrepancies, space loss, and eruption disturbances may improve occlusal development and simplify subsequent treatment phases [4,6].

Objective assessment of treatment effectiveness requires standardized indices, as clinical judgment alone may vary between examiners. The use of validated tools such as the Peer Assessment Rating (PAR) index allows reproducible quantification of malocclusion severity and treatment outcomes, facilitating audit, comparison, and research standardization [7]. Specifically, the PAR index provides a weighted summary score of occlusal deviation by assessing key components such as anterior and posterior segment alignment, buccal occlusion, overjet, overbite, and midline discrepancies, allowing comparison between serial records and objective estimation of treatment improvement according to predefined standards [7]. Advances in digital workflows and automated measurement systems may further enhance reliability and consistency in orthodontic outcome assessment [8].

With increasing demand for aesthetic and comfortable alternatives to conventional appliances, clear aligner therapy has expanded into the pediatric population. Invisalign^®^ First was introduced in 2018 as a system specifically designed for early mixed dentition, incorporating features such as eruption compensation and digitally planned dentoalveolar expansion within the ClinCheck^®^ workflow [9,10]. Therefore, this retrospective cohort study was designed as a continuation and expansion of the previously published investigation by [4], applying the same methodological framework to a larger sample in order to strengthen the evidence regarding interceptive treatment with Invisalign^®^ First. In addition to replicating the original digital and cephalometric assessment protocol, the present study incorporates an objective evaluation of occlusal outcomes using the Peer Assessment Rating (PAR) index and includes stability analysis during the retention phase. Accordingly, the primary aim of this study was to describe longitudinal changes in occlusal outcomes after Phase I treatment with Invisalign^®^ First in early mixed dentition, using the Peer Assessment Rating (PAR) Index from baseline (T0) to post-treatment (T1) and retention follow-up (T2). The secondary aims were to assess changes in overall case complexity and specific occlusal parameters from baseline to post-treatment using an Align^®^ protocol-derived complexity index and digital/cephalometric measurements, and to describe the maintenance of occlusal outcomes during retention.

## 2. Materials and Methods

### 2.1. Study Design and Sample Eligibility Criteria

This study was designed as a retrospective evaluation of Phase I (interceptive) clear aligner therapy with Invisalign^®^ First (Align Technology, San Jose, CA, USA) in children in the early mixed dentition, based on records collected at private practice centers treated by Pinho T. between early 2019 and late 2025. Three assessment time points were defined: T0 (baseline), T1 (end of Phase I treatment), and T2 (retention), with T2 operationally defined as a follow-up assessment performed at least 18 months after T1.

Given that two indices were applied with different time-point requirements, two nested analytic samples were considered. The primary longitudinal dataset consisted of 55 patients with complete digital records at T0, T1, and T2, enabling PAR Index scoring, which is based exclusively on occlusal parameters derived from digital models. Baseline skeletal maturation was assessed in all included patients using the cervical vertebral maturation (CVM) method on the initial lateral cephalogram. All patients were classified as CS1–CS2, with seven patients classified as CS1, indicating a comparable prepubertal skeletal maturation stage at treatment onset [11].

Within this longitudinal cohort of 55 patients, a nested subsample of 47 patients with complete T0–T1 cephalometric records was identified for application of the Align^®^ protocol-derived trait-based complexity index [4]. Cephalometric evaluation was limited to T0–T1 comparisons, as lateral cephalograms were obtained only when clinically justified according to radiographic selection guidelines; therefore, T2 cephalometric records were not routinely acquired in order to avoid unnecessary radiation exposure. Patients were selected based on eligibility criteria consistent with interceptive mixed-dentition protocols. Eligible cases comprised children in early mixed dentition treated with Invisalign^®^ First in both arches, with complete baseline and end-of-treatment intraoral digital scans, and who presented with at least one malocclusion trait requiring intermediate-to-high complexity correction according to an adaptation of the Align^®^ protocol. Patients with previous or concomitant orthodontic treatment, craniofacial malformations (including cleft lips and/or palate), history of dental trauma, oral neoformations, or other oral cavity pathologies were excluded.

Part of the sample included in the present study has been previously reported in earlier publications [4,6], addressing different research questions and outcome measures. The present study expands the dataset by incorporating longitudinal occlusal assessment (PAR Index) and stability analysis. This was a single-arm retrospective study without an untreated or alternative treatment control group and therefore does not allow causal inference.

### 2.2. Ethical Considerations

The study was approved by the Ethics Committee of the University Institute of Health Sciences (IUCS-CESPU), under reference 43/CE-IUCS/2025. The parents and legal guardians of all participating children were fully informed of the study procedures, and written informed consent was obtained prior to data collection and analysis.

### 2.3. Intervention Protocol and Records Acquisition

All patients underwent Phase I interceptive treatment with Invisalign^®^ First. They were instructed to wear the aligners 20–22 h a day, removing them only for eating and oral hygiene. Each aligner was designed to deliver staged movements consistent with standard aligner biomechanics (approximately 0.25 mm of translation and 1° of rotation per aligner) [12]. Aligner replacement followed a 7-day change protocol; in more complex cases requiring highly sequenced movements (typically involving >50 aligners in the initial series), the change frequency could be increased to twice weekly if compliance was confirmed and aligner fit was adequate. Clinical follow-up visits were scheduled every 4 weeks. The active Phase I treatment period (T0–T1) was approximately 18 months.

The use of intermaxillary elastics as adjunctive mechanics should be considered as part of the overall treatment protocol rather than the effect of aligners alone. In addition, in selected Class II growing patients, mandibular advancement (MA) features integrated within the Invisalign^®^ system were used as part of the interceptive treatment protocol, as previously described [6].

Intermaxillary elastics were prescribed as auxiliaries from the beginning of treatment in moderate-to-complex posterior crossbite cases and in complex Class II/Class III sagittal relationships; in Class II division 2 situations, elastics were introduced only after sufficient overjet had been created. The last set of aligners, when needed, was prescribed near the end of Phase I to finalize the objectives or to serve as a retention strategy, and were typically worn only during sleep.

Clinical records were obtained at T0 (baseline) and T1 (end of active Phase I) for all patients, and at T2 (retention) when available. Digital records were acquired using an iTero^®^ Element 5D Plus scanner (Align Technology, Tempe, AZ, USA) and exported as STL models, which were subsequently analyzed within the ClinCheck^®^ workflow (ClinCheck Pro 6.0 software, Align Technology Inc., San José, CA, USA). Planned and achieved movements were quantified using ClinCheck^®^ built-in measurement outputs, including the planned movement table, millimetric ruler tools, and arch-width tables. Intraoral photographs were additionally reviewed to confirm the occlusal registration and bite position of the digital models.

Where applicable, lateral cephalometric radiographs were obtained at T0 and T1 and analyzed using cephalometric software NemoStudio^®^ Software V.25.0.0.0 (Nemotec, Madrid, Spain), enabling the assessment of skeletal and dentoalveolar changes alongside the digital model-based measurements. Lateral cephalometric radiographs were not routinely obtained at T1 or T2, and were prescribed only when clinically justified, in accordance with radiographic selection guidelines and the principle of keeping exposure as low as reasonably achievable (ALARA) [13].

### 2.4. Outcome Measures and Indices

#### 2.4.1. Peer Assessment Rating (PAR) Index: Occlusal Outcome and Stability Assessment

Occlusal outcome was quantified longitudinally using the Peer Assessment Rating (PAR) Index in the full cohort (*n* = 55). This established metric measures the degree of deviation from an optimal occlusion and objectively evaluates orthodontic treatment outcome by comparing pre-treatment (T0), post-treatment (T1), and retention follow-up records (T2; ≥18 months after T1)**.** The PAR Index was operationalized through the weighted British PAR system, ensuring uniformity and comparability across cases [14].

The PAR Index comprises five principal components: maxillary and mandibular right/left/anterior segments, right and left buccal occlusion, overjet, overbite, and midline discrepancies [15]. PAR component data were extracted from digital models, using standardized digital measurement protocols. Overjet was obtained from the digital records (STL/ClinCheck^®^ measurement outputs), and cephalometric values were used to confirm only when available. All scoring was conducted blindly, with the evaluator unaware of whether models corresponded to T0, T1, or T2.

Weighted scoring followed the British weighting scheme: the right, left, and anterior segments (both arches) and lateral buccal occlusions (right and left) were assigned a weight of 1; overjet was weighted by 6, overbite by 2, and midline discrepancies by 4. The final weighted PAR score was calculated as the sum of all weighted components [14]. Percentage improvement was computed as: %PAR = (PAR T0 − PAR Tx)/PAR T0 × 100%, enabling standardized comparison of change over time [6] between T0–T1 and T0–T2.

Cases were categorized by percentage improvement into worse or no change (<30% improvement), improved (30–70% improvement), and greatly improved (≥70% improvement), providing a clinically interpretable classification of treatment effectiveness [14]. In the present study, PAR scoring is applied across the defined timepoints to quantify occlusal change after active treatment and during follow-up, using the same weighted framework and categorical thresholds to support longitudinal assessment. The PAR Index reflects occlusal changes over time but does not allow differentiation between treatment effects and changes related to growth and dental development, particularly in mixed dentition.

#### 2.4.2. Align^®^ Protocol-Based Complexity Index and Global Complexity Score (Nested Subsample, *n* = 47)

In a nested subsample of 47 patients with complete T0-T1 cephalometric records, treatment complexity and trait predictability were assessed using an index derived from the Align^®^ mixed dentition protocol [4].

The index is a literature-based mixed dentition complexity framework adapted from clinical recommendations associated with the Align^®^ protocol, which classifies interceptive malocclusion traits as predictable (1), intermediate (2), or difficult (3), integrating digital treatment planning data (ClinCheck^®^), intraoral scans (STL models), standardized clinical records, and cephalometric analysis (T0–T1). The individual T0–T1 cephalometric and occlusal measurements used to characterize the cephalometric subsample are provided in Table S1 in the supplementary materials section.

The evaluated traits included molar derotation, dentoalveolar expansion, space recovery, molar sagittal malocclusion, posterior crossbite, open bite, midline discrepancy, crowding, and skeletal involvement. Skeletal involvement was dichotomously weighted within the global complexity score. This classification reflects the presence of skeletal discrepancy rather than its direction, as both hypo- and hyperdivergent patterns are categorized as severe skeletal involvement. A severe skeletal component (+3) was assigned when cephalometric analysis revealed a severe sagittal discrepancy (ANB ≥ 7° or ANB ≤ 0°) and/or a Severe vertical discrepancy (FMA ≥ 30° or FMA < 20°). Cases not meeting these thresholds were assigned a skeletal score of 0 (ANB 1–6° and FMA > 20° and <30°), indicating the absence of marked skeletal discrepancy (Table 1). This threshold was defined considering the characteristics of a growing population, in which ongoing craniofacial development, particularly mandibular growth, may influence sagittal relationships over time and allow for spontaneous improvement of mild discrepancies. Cases not meeting these severe skeletal thresholds were assigned a skeletal component score of 0 in this index; however, this should not be interpreted as normal skeletal relationships, but rather as the absence of a marked skeletal discrepancy according to the operational criteria adopted for this study.

A global quantitative complexity score was calculated by summing the individual trait scores (range 1–3 per trait) as shown in (Table 2). The global complexity score was obtained by summing ordinal trait scores assigned according to the Align-based protocol. This composite score was used as an ordered clinical measure to summarize overall case burden and to compare complexity changes within the same cohort, rather than as a continuous interval-scale variable. Based on cumulative burden, cases were categorized as predictable (≤3), moderate (4–8), or severe (>8). Also, the percentage of improvement in complexity was calculated using the following formula: Improvement % = (T0 − T1)/T0 × 100%.

This index enabled quantitative assessment of trait-based complexity reduction between T0 and T1 within the cephalometric subsample (*n* = 47), complementing the longitudinal occlusal outcome evaluation performed with the PAR Index in the full cohort (*n* = 55); however, because it is derived from the Align^®^ clinical protocol and has not been externally validated, its generalizability and reproducibility outside this specific framework may be limited.

### 2.5. Measurement Reliability and Potential Bias

All measurements were performed by a calibrated examiner using standardized digital protocols and subsequently reviewed by a second examiner. A standardized verification process was used to enhance accuracy and reproducibility; however, measurement error cannot be completely excluded.

### 2.6. Sample Selection Considerations

Only patients with complete longitudinal records (T0, T1, and T2) were included, which may have resulted in a sample with higher compliance and more consistent follow-up. Therefore, the findings may not be fully generalizable to all treated patients.

### 2.7. Statistical Analysis

Data analysis was performed using IBM^®^ SPSS^®^ (Statistical Program for Social Sciences) software, version 29.0 for Windows (IBM Corp., Armonk, NY, USA), and Microsoft Excel^®^ (Microsoft Excel for Microsoft 365, Version 2206, Microsoft Corporation, Redmond, WA, USA) was used for data organization and graphical reporting. Descriptive statistics were generated, including frequencies and percentages, means, medians, standard deviations, and minimum and maximum values. The Shapiro–Wilk test was used to assess the sample’s normality. Because the data did not follow a normal distribution, nonparametric tests were applied. The Wilcoxon signed-rank test was used to compare cephalometric metrics (i.e., overjet, overbite, ANB, FMA, and IMPA) before and after treatment, and to compare dental component measurements (molar derotation, dentoalveolar expansion, space recovery, and crowding) before and after treatment. To compare occlusal complexity before, after, and during the retention phase, Friedman’s test and the Wilcoxon signed-rank test were used. The significance level was set at 0.05.

## 3. Results

### 3.1. Descriptive Statistics

In the full longitudinal cohort analysed using the PAR index (*n* = 55), 55 Caucasian children aged 6–11 years were included (mean age 8.5 ± 1.1 years), comprising 33 females (60.0%) and 22 males (40.0%). According to the cervical vertebral maturation method, 12.7% of patients were classified as cervical stage 1 (CS1), whereas the remaining 87.3% were classified as cervical stage 2 (CS2) [11]. The number of additional aligners prescribed per patient ranged from 1 to 4 (mean 2.6 ± 0.8). The retention period ranged from 18 to 54 months, with a mean follow-up of 27.6 ± 12.0 months. At T2, all patients had transitioned to permanent dentition, and full eruption of the posterior support zone was observed in 100% of cases.

Within this cohort, a nested subsample of 47 patients with complete T0–T1 cephalometric records was evaluated using the modified Align^®^ protocol-based complexity index [4]. This subsample comprised 26 females (55.3%) and 21 males (44.7%), with a mean age of 8.6 ± 1.1 years. The number of additional aligners ranged from 1 to 4 (mean 2.6 ± 0.87).

### 3.2. Case Series for the PAR Index

Table 3 presents the results regarding changes in occlusal complexity, assessed using the Peer Assessment Rating (PAR) index, throughout interceptive aligner treatment. To compare the reduction in PAR between the T0–T1 interval (before vs. after aligner therapy) and the T1–T2 interval (from the end of aligner therapy to the retention phase), the Wilcoxon signed-rank test was used. In the 55 cases analyzed, the reduction in PAR was greater during T0–T1 than during T1–T2, with all reductions corresponding to negative ranks (*n* = 55); no positive ranks or ties were recorded. These differences were statistically significant (*p* < 0.001), indicating that the greatest reduction in occlusal complexity occurred during active aligner treatment, whereas the retention phase was characterized by a significantly smaller decrease in PAR. Across the 55 cases, the overall reduction in complexity ranged from 31.8% to 100%, with a mean reduction of 75.82 ± 15.25%.

Occlusal complexity was assessed at three time points: before the start of aligner treatment (T0), after completion of treatment (T1), and during the retention phase (T2) (Table 4). A marked reduction in median occlusal complexity values was observed over time, decreasing from 22.0 (IQR 17.0–30.0) at T0 to 6.0 (IQR 1.0–8.0) at T1 and 7.0 (IQR 2.0–10.0) at T2, indicating a marked reduction in occlusal complexity, with values remaining relatively stable during the follow-up period.

The Friedman test revealed statistically significant differences across the three time points (*p* < 0.001). Post hoc pairwise comparisons with Bonferroni correction demonstrated statistically significant differences between T0 and T1 (*p* < 0.001) and between T0 and T2 (*p* < 0.001), but not between T1 and T2 (*p* = 0.546). These findings indicate that occlusal complexity decreased substantially over time, with no statistically significant differences between post-treatment and follow-up assessments.

### 3.3. Case Series for the Align^®^ Based Protocol Index Used in the Previous Study [4]

Table 5 summarizes the dental component molar derotation, dentoalveolar expansion, space recovery, and crowding measurements, as well as cephalometric outcomes assessed before and after treatment with aligners. For the dental component, molar derotation of teeth 16 and 26 showed that the achieved movements were generally lower than the initially planned values, indicating partial expression of the prescribed movements. Dentoalveolar expansion in both upper and lower arches decreased substantially from the initially planned to post-treatment measurements. Similarly, space recovery and crowding in both arches were notably reduced after treatment.

Planned movements represent intended tooth displacement within the digital setup, whereas achieved values reflect clinical outcomes; differences between both should be interpreted as a degree of expression rather than direct treatment success or failure.

Cephalometric analysis revealed an increase in overbite and a reduction in overjet at the end of treatment. Skeletal parameters exhibited modest changes, with slight reductions in ANB and FMA and increases in IMPA values from baseline to the end of treatment.

Table 6 reports the results of the Wilcoxon signed-rank tests comparing initially measured and final measured cephalometric variables. A statistically significant reduction in the ANB angle was observed from initial measurement to the end of treatment (*p* = 0.043), with a predominance of negative ranks, indicating lower ANB values at the final assessment in most patients. The IMPA showed a significant increase from initial to final measurements (*p* = 0.004), with positive ranks predominating, reflecting proclination of the lower incisors over the course of treatment. The FMA also demonstrated a significant change between initial and final measurements (*p* = 0.012), with more negative ranks, indicating an overall reduction in FMA at the end of treatment. Overbite increased significantly from the initial to the final evaluation (*p* = 0.004), as evidenced by the predominance of positive ranks. Overjet exhibited the most pronounced change, with a highly significant reduction from the initial to the final measurements (*p* < 0.001), supported by the large number of negative ranks.

Table 7 reports the results of Wilcoxon signed-rank tests comparing initially measured and final measured dental-component outcomes, including molar derotation, dentoalveolar expansion, space recovery, and crowding. Regarding molar derotation, both the maxillary right first molar (tooth 16) and the maxillary left first molar (tooth 26) showed a predominance of negative ranks at the end of treatment, indicating a statistically significant reduction in rotational angulation (*p* < 0.001 for both). For dentoalveolar expansion, negative ranks predominated for both maxillary and mandibular arch widths after treatment, demonstrating a statistically significant increase in transverse expansion (*p* < 0.001 for both). With respect to space recovery, negative ranks predominated in both the maxillary (*p* < 0.001) and mandibular arches (*p* = 0.012), indicating a significant increase in available space for dental alignment. Finally, for the crowding malocclusion, negative ranks predominated in both arches (maxillary: *p* < 0.001; mandibular: *p* < 0.001), indicating a statistically significant reduction in crowding following treatment.

**Figure 1:**
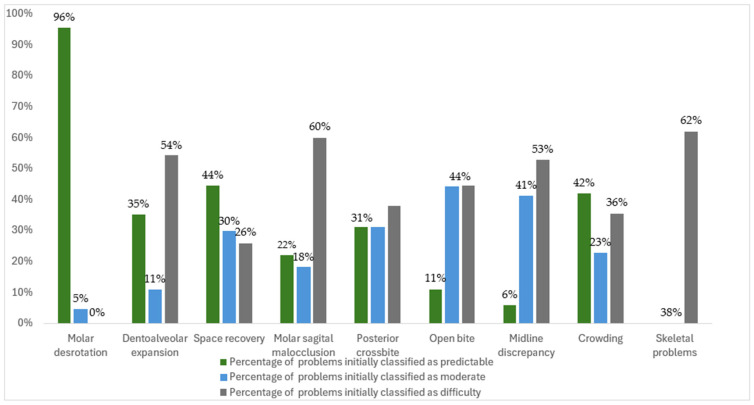
Distribution of malocclusion traits and their complexity classification before the first aligner series.

**Figure 2:**
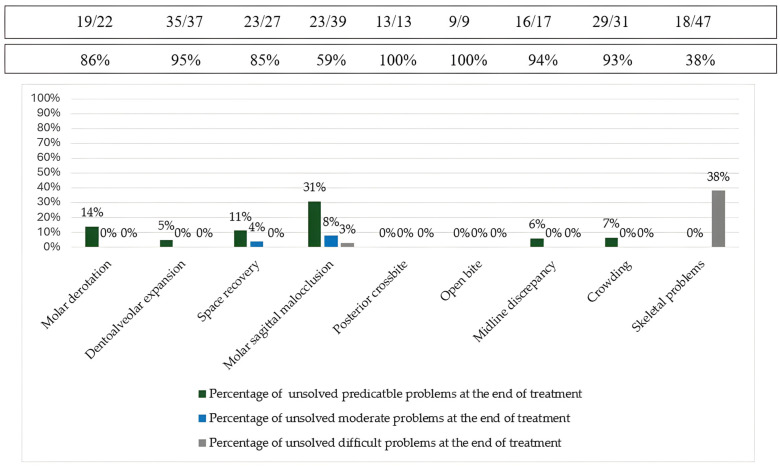
Percentages of malocclusion traits that were not corrected at the end of treatment according to the problem classification.

Figure 1 shows the global complexity classification of the 47 cases prior to the first aligner series. Within this sample, 22 cases presented molar derotation, 37 dentoalveolar expansion, 27 space recovery, 39 molar sagittal malocclusion, 13 posterior crossbite, 9 open bite, 17 midline discrepancy, 31 crowding, and 29 skeletal problems were identified in 29 of 47 patients (62%); the percentages displayed in Figure 2 were calculated relative to the number of problems recorded within each category. Most traits were classified as moderate-to-difficult, except for molar derotation, which was considered predictable in 96% of cases. In contrast, dentoalveolar expansion, space recovery, molar sagittal malocclusion, crowding, and skeletal problems showed low proportions of predictable and moderate movements and high proportions of difficult cases.

Figure 2 summarizes post-treatment outcomes by complexity grade. Overall, aligner therapy was effective in correcting the evaluated malocclusion traits, with success rates exceeding 85% for molar derotation, dentoalveolar expansion, space recovery, midline discrepancy, and crowding, reaching 100% for correction of posterior crossbite and open bite. In contrast, molar sagittal malocclusion and skeletal problems showed more modest success rates (59% and 38%, respectively). Across the 47 cases, the reduction in complexity ranged from 0% to 100%, with a mean reduction of 79.49 ± 22.43%.

## 4. Discussion

The findings of this study should be interpreted within the limitations of a single-arm retrospective design. In the absence of an untreated control group, it is not possible to distinguish the relative contribution of observed treatment-period changes from normal growth and dental development, particularly in a growing population. Therefore, the results should be interpreted as observed longitudinal changes rather than definitive evidence of treatment efficacy. Additionally, the inclusion of cases previously analysed under different study designs allows a more comprehensive longitudinal interpretation of treatment effects across multiple outcome domains [4,6].

This retrospective cohort study builds on the previously published case series [4] by applying the same trait-based complexity framework to a larger sample and integrating a standardized occlusal outcome and longitudinal maintenance during dental development using the PAR Index [7,12]. By combining trait-based complexity reduction (*n* = 47) with longitudinal occlusal outcome assessment (*n* = 55), this design enhances both methodological continuity and descriptive longitudinal assessment of treatment-related changes.

Consistent with the 2022 findings [4], interceptive treatment with Invisalign^®^ First was associated with a substantial reduction in malocclusion complexity, particularly in dentoalveolar domains. The baseline distribution showed a predominance of moderate-to-severe cases, consistent with the case selection criteria for interceptive treatment with aligners in mixed dentition, where this modality is preferentially indicated for malocclusion traits of moderate-to-severe complexity.

In contrast, milder and more predictable discrepancies may often be managed with simpler interceptive approaches, including bite ramps or myofunctional appliances (e.g., Trainer for Kids), aimed primarily at correcting functional imbalances or guiding occlusal development. Although clear aligners can also be used in these situations, the present treatment protocol prioritizes their use in moderate-to-severe cases, where greater dentoalveolar correction is required.

Following treatment, a marked shift toward lower complexity categories was observed, indicating a substantial reduction in occlusal burden during Phase I treatment [6,10].

Trait-level analysis provides further mechanistic insight. Dentoalveolar objectives, including crowding, midline discrepancies, posterior crossbite, open bite, and transverse development, demonstrated high correction rates, exceeding 85% in most domains and reaching 100% in posterior crossbite and open bite correction. These high correction rates observed in dentoalveolar traits further support the clinical applicability of aligner-based interceptive protocols in moderate-to-severe cases [4,6,9,10,15,16,17], as the controlled force systems generated by staged aligner activation may help explain the observed transverse development and alignment changes; this interpretation is supported by recent mixed-dentition studies showing that Invisalign^®^ First and clear aligners can produce significant dentoalveolar transverse changes and measurable arch-expansion predictability, although the magnitude of expression may vary and refinements are often needed to improve predictability, particularly in growing patients where dentoalveolar adaptability is enhanced [9,18]. This interpretation is supported by recent mixed-dentition studies showing that clear aligners can produce measurable transverse dentoalveolar changes and arch-development effects, although the degree of expansion expression may vary between arches, tooth groups, and refinement stages [19,20,21].

In contrast, sagittal molar relationships and skeletal discrepancies exhibited more limited responsiveness, even when adjunctive approaches such as intermaxillary elastics and, in selected cases, mandibular advancement protocols were incorporated. Previous analysis of a subsample treated with mandibular advancement [6] demonstrated that, although functional correction can be achieved in growing patients, treatment response remains variable and dependent on individual growth potential. In that study, a small number of cases did not achieve full sagittal correction, highlighting the inherent biological variability and limitations of growth-modifying approaches. This is consistent with the present findings, where sagittal and skeletal components showed lower correction rates compared to dentoalveolar domains, reinforcing that interceptive aligner therapy primarily achieves dentoalveolar correction, while skeletal discrepancies may require additional or subsequent treatment phases.

Although statistically significant changes were observed in some cephalometric parameters (ANB reduction, *p* = 0.043; IMPA increase, *p* = 0.004; FMA change, *p* = 0.012), the skeletal component of the complexity index often remained unchanged. This may partially reflect a methodological characteristic of the index rather than the absence of skeletal improvement, as the skeletal component is dichotomously scored (+3) when values exceed predefined thresholds (ANB ≥ 7° or ≤0°, FMA ≥ 30° or <20°). Consequently, cases may show quantitative cephalometric improvement while remaining within the same severity category. Nevertheless, the reductions observed in ANB and FMA should be interpreted cautiously, as these changes may reflect a combination of dentoalveolar adaptation and normal growth rather than direct skeletal modification [22]. These findings are consistent with the observations reported in 2022 and 2025 [4,6] and reinforces the biological limitation inherent to Phase I therapy: aligners are highly effective in dentoalveolar correction but cannot fully resolve growth-dependent skeletal disharmonies in all patients [2,3,23]. This interpretation is consistent with recent systematic reviews in growing patients, which highlight that current evidence remains heterogeneous and that dentoalveolar outcomes are more consistently documented than skeletal modifications [24].

Importantly, residual skeletal classification should not be interpreted as treatment failure. Rather, it reflects the intended scope of interceptive care: reduction in occlusal severity and functional imbalance during growth, while acknowledging that definitive skeletal correction may require subsequent orthopaedic or comprehensive treatment phases [3,6].

A major advancement of the present study lies in the integration of the PAR Index for longitudinal outcome assessment. According to [7], a PAR reduction exceeding 70% is indicative of high-standard orthodontic treatment. The mean PAR reduction of 75.82% in this cohort surpasses this benchmark, indicating substantial occlusal improvement during the treatment period.

Furthermore, the absence of statistically significant differences between T1 and T2 (*p* = 0.546) suggests maintenance of occlusal outcomes during a minimum 18-month follow-up period. Although a slight increase in median PAR values was observed at T2, the overall reduction relative to baseline remained clinically meaningful. These findings are consistent with stability trends reported in aligner-treated cohorts [25,26] and represent a methodological advancement over the 2022 publication [4], in which longitudinal occlusal stability was not assessed. In growing patients, true orthodontic stability cannot be definitively established, as ongoing growth and dental eruption may influence occlusal indices over time. Therefore, stability outcomes should be interpreted with caution within the context of growth.

Collectively, the data indicate that interceptive aligner therapy was associated with clinically relevant improvements, particularly in dentoalveolar domains, with statistically and clinically significant reductions in occlusal complexity, and with maintenance of occlusal outcomes during the observation period, which should be interpreted within the context of growth and study design limitations. Within the clinical context of mixed dentition, these findings suggest that aligner-based Phase I therapy may be associated with reduced malocclusion severity and may facilitate subsequent orthodontic management when needed, although these observations should be interpreted within the context of growth and study design limitations.

### 4.1. Study Strengths and Limitations

This study presents several strengths. First, it maintains strict methodological continuity with the previously validated complexity framework [4], enhancing reproducibility. Second, the dual-index design, combining a trait-based Align^®^ protocol-derived complexity model with a validated global occlusal index (PAR), provides a multidimensional assessment of both predictability and longitudinal occlusal outcomes. Third, structured digital records and standardized measurement tools contribute to internal consistency. Although the PAR index presents recognized limitations in early mixed dentition, particularly regarding contact-point displacement involving deciduous teeth, its use remains applicable in transitional mixed dentition with erupted permanent incisors and first molars. Importantly, longitudinal stability assessment at T2 was performed after transition to permanent dentition, with complete eruption of the posterior support zones in all patients.

However, limitations must be acknowledged. The retrospective design restricts causal inference and introduces potential selection bias, particularly due to the inclusion of patients with complete longitudinal records. The absence of an untreated control group represents a major limitation, as spontaneous improvements related to growth and dental development cannot be excluded.

Clinical heterogeneity in mixed dentition, where eruption dynamics and growth progression are variable, limits the ability to isolate trait-specific treatment effects independent of natural development.

Compliance-related bias remains a relevant factor in aligner therapy, given the dependence on patient cooperation. Although objective wear-time monitoring was not available, aligner tracking and patient compliance were routinely verified during regular clinical follow-up visits through assessment of aligner fit and tracking.

Additionally, cephalometric evaluation was not routinely performed during retention, consistent with ALARA-guided radiographic protocols [11]. While ethically justified, this limits long-term skeletal stability assessment beyond occlusal outcomes.

Although all measurements were performed using a standardized protocol and verified by a second examiner, formal intra- and inter-examiner reliability testing was not conducted, which may affect internal validity. Furthermore, the use of a proprietary Align^®^-derived complexity index may limit generalizability, as its validity outside the ClinCheck^®^ environment has not been independently established.

### 4.2. Clinical Implications

The findings support the clinical applicability of Invisalign^®^ First as an interceptive modality in moderate-to-severe mixed dentition cases. Clinicians should anticipate a favourable dentoalveolar response, particularly transverse development and alignment, while maintaining realistic expectations regarding skeletal modification.

The integration of PAR-based assessment supports the use of standardized outcome measures in interceptive aligner research, facilitating benchmarking and cross-study comparison [7,8].

Overall, aligner-based interceptive therapy may represent a valuable clinical option for contributing to a reduction in malocclusion severity during mixed dentition and facilitating subsequent orthodontic management when needed, although these observations should be interpreted within the context of growth and study design limitations.

## 5. Conclusions

Dentoalveolar objectives demonstrated high correction rates, whereas skeletal and sagittal discrepancies showed statistically significant but biologically limited modification, consistent with the scope of Phase I therapy.

The integration of PAR assessment showed a mean reduction exceeding 75%, indicating substantial reductions in weighted occlusal scores over a minimum 18-month follow-up period during dental development.

Overall, interceptive treatment with Invisalign^®^ First showed longitudinal reductions in occlusal complexity and favorable dentoalveolar correction rates in early mixed dentition, with maintenance of favorable occlusal outcomes during follow-up as patients transitioned from mixed to permanent dentition. These findings should be interpreted as descriptive longitudinal observations rather than definitive evidence of causal treatment effects or true long-term occlusal stability, particularly given the absence of a control group and the influence of growth and dental development.

Further controlled prospective studies are required to clarify the specific contribution of aligner therapy in interceptive orthodontic treatment.

## Figures and Tables

**Table 1:** Reference values for the classification of the malocclusion traits into predictable, intermediate, or difficult corrections based on Align^®^ recommendations, with the respective alterations.

Malocclusion Traits /Required Movements	Type of Measurement	Predictability with Aligners		
		Predictable (1)	Intermediate (2)	Difficult (3)
Molar derotation (teeth 16 & 26)	Initially planned by ClinCheck^®^	15–30°	>30–40°	>40°
Dentoalveolar Expansion	The difference between the initially ClinCheck^®^ planned and experimentally measured (STL)	3–4 mm and negative molar torque	>4–6 mm and negative molar torque	>6 mm or >4 mm and Positive molar torque and Skeletal compression
Space to recover	The difference between the initially planned by ClinCheck^®^ and experimentally measured (STL)	2–4 mm	>4–6 mm	>6 mm
Molar sagittal malocclusion	ClinCheck^®^ and ANB ceph	Class II Functional/Dental ≤½ cusp or molar ectopic eruption)	Mild skeletal ½ cusp Tendency for Class III	Skeletal Class II (complete) ≥1 cusp or Class III
Posterior crossbite	Intraoral Photographs STL	Crossbite only on deciduous teeth	Crossbite in deciduous/ permanent teeth and Negative molar torque	Crossbite in permanent teeth and Positive molar torque and Skeletal compression
Open bite	Initially planned by ClinCheck^®^ and FMA	Posterior intrusion: <0.5 mm and/or Anterior extrusion: <2.5 mm	Posterior intrusion: >0.5–1 mm and/or Anterior extrusion: >2.5–3.5 mm	Hyperdivergent Posterior intrusion: >1 mm and/or Anterior extrusion: >3.5 mm
Midline discrepancy	Initially experimentally Measured STL	2 mm	>2 and <3 mm	≥3 mm
Crowding	Required space initially experimentally measured and required incisor rotation planned by ClinCheck^®^	3–6 mm	>6–8 mm or Lateral incisors: 30–40◦ or Central incisors 40–50◦	>8 mm or Lateral incisors: >40◦ or Central incisors: >50◦
Skeletal problem	Cephalometric Analysis ANB/FMA			ANB ≥ 7° or ANB ≤ 0° and/or FMA ≥ 30° or FMA < 20°

Caption: Skeletal score 0 was assigned when ANB values were between 1° and 6° and FMA values were >20° and <30°, indicating the absence of marked skeletal discrepancy.

**Table 2:** Qualitative evaluations of each objective and the global complexity classification of every case.

	Age & Sex	Molar Derotation (*n* = 22)	Dentoalveolar Expansion (*n* = 37)	Space to Recover (*n* = 26)	Molar Sagittal Class (*n* = 40)	Posterior Crossbite (*n* = 13)	Open Bite (*n* = 9)	Midline Discrepancy (*n* = 17)	Crowding (*n* = 31)	Skeletal Problem (*n* = 42)	TOTAL
**Case 1**	9 M	0	3	2	2	3	0	3	3	3	19
**Case 2**	8 F	0	0	2	0	1	3	0	3	3	12
**Case 3**	9 F	0	1	1	3	0	0	0	1	3	9
**Case 4**	9 F	1	2	2	0	0	0	0	1	3	11
**Case 5**	9 F	1	0	2	2	0	0	0	3	3	11
**Case 6**	8 M	1	3	0	3	0	0	0	0	3	10
**Case 7**	9 M	1	0	0	3	0	0	0	0	3	7
**Case 8**	9 F	1	1	0	3	0	1	0	0	0	6
**Case 9**	8 F	1	3	3	1	0	3	0	2	3	16
**Case 10**	9 F	1	2	1	3	2	0	3	0	0	12
**Case 11**	8 F	0	0	0	3	1	0	0	0	0	4
**Case 12**	8 M	0	1	1	2	2	0	0	1	3	10
**Case 13**	9 M	0	3	3	0	2	0	3	3	0	14
**Case 14**	11 M	1	3	0	1	0	0	0	2	3	10
**Case 15**	9 M	0	3	3	1	0	0	0	3	0	10
**Case 16**	10 F	2	3	3	2	0	0	3	1	3	17
**Case 17**	10 F	1	3	0	3	0	0	1	0	3	11
**Case 18**	8 F	0	3	0	3	0	0	0	0	0	6
**Case 19**	9 F	0	1	1	2	0	0	3	0	3	10
**Case 20**	8 M	1	1	2	3	0	0	0	1	3	11
**Case 21**	10 M	0	1	0	2	0	0	0	0	3	6
**Case 22**	8 F	1	1	2	3	0	0	0	1	3	13
**Case 23**	8 M	0	3	1	1	2	0	2	0	0	9
**Case 24**	7 M	0	3	0	1	3	0	3	0	0	10
**Case 25**	10 M	0	1	1	0	0	0	0	2	3	7
**Case 26**	9 F	1	0	0	3	0	3	0	2	3	12
**Case 27**	8 M	0	3	1	1	0	0	2	1	0	8
**Case 28**	7 F	1	3	0	3	0	0	2	2	3	14
**Case 29**	10 M	0	3	0	3	3	0	0	1	0	10
**Case 30**	7 F	0	0	1	3	0	0	0	3	0	7
**Case 31**	9 F	1	1	3	1	0	0	3	3	3	15
**Case 32**	9 F	0	1	0	2	3	0	2	0	0	8
**Case 33**	7 F	1	3	1	3	1	3	0	3	3	18
**Case 34**	10 F	1	1	2	0	0	0	2	2	0	8
**Case 35**	11 M	0	3	3	1	0	0	0	3	0	10
**Case 36**	6 M	1	3	0	0	0	2	0	1	3	10
**Case 37**	9 F	0	3	0	3	0	0	2	1	3	12
**Case 38**	8 M	0	0	0	3	0	2	0	0	3	8
**Case 39**	9 F	0	2	1	3	0	0	0	1	3	10
**Case 40**	9 F	1	1	0	3	0	0	3	0	3	11
**Case 41**	9 F	1	0	1	3	0	2	0	1	0	8
**Case 42**	8 M	0	0	0	3	3	0	0	0	0	6
**Case 43**	9 M	1	2	0	0	1	0	2	0	0	6
**Case 44**	8 F	1	1	0	3	0	0	0	2	3	10
**Case 45**	7 M	0	3	3	3	0	0	3	3	3	18
**Case 46**	8 M	0	0	2	1	0	0	0	3	3	9
**Case 47**	6 F	0	3	1	3	0	2	0	1	0	10

Caption: F: female; M: male; objectives to be achieved: Predictable (1); Intermediate (2), Difficult (3). Case global complexity: 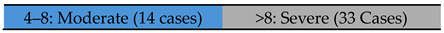

**Table 3:** Comparison of PAR index reduction between active treatment and retention phases (Wilcoxon signed-rank test).

		*n*	Mean Rank	Z	*p*
PAR index after (T1–T2) vs. PAR index before (T0–T1)	Negative Ranks	55 ^†^	28.00	−6.455	<0.001
	Positive Ranks	0	0.0		
	Ties	0			
	Total	55			

**Caption:** ^†^ Negative ranks represent situations where the final variable measurement is lower than the initial variable measurement; positive ranks represent situations where the final variable measurement is greater than the initial variable measurement; and ties represent situations where the initial values are equal to the final values.

**Table 4:** Comparison of occlusal complexity between T0, T1, and T2 (Friedman test and Bonferroni corrected pairwise comparisons).

					*T*0 *vs. T*1	*T*0 *vs. T*2	*T*1 *vs. T*2
	*n*	*Median* [*IQR*]	χ^2^	*p*	*p*	*p*	*p*
Total after T0	55	22.0 [17.0, 30.0]	88.72	<0.001	<0.001	<0.001	0.546
Total after T1	55	6.0 [1.0, 8.0]					
Total after T2	55	7.0 [2.0, 10.0]					

Caption: Friedman test results. Pairwise comparisons between time points were performed using the Wilcoxon Signed-Rank test with Bonferroni correction. Data are presented as medians and interquartile ranges (IQR).

**Table 5:** Descriptive statistics regarding derotation, dentoalveolar expansion, space recovery, and crowding measurements for the teeth presenting more prominent movements to achieve, as well as cephalometry metrics for all patients.

	Mean	SD	Median	Min	Max	*n*
Dental Component						
Molar derotation (°)						
Initially planned, 16	13.53	7.39	12.80	0.6	35.9	47
Planned at the end, 16	2.83	3.98	1.60	0.0	16.5	47
Dif. (initially planned), 16	10.70	8.30	10.50	−13.50	35.10	47
Initially planned, 26	10.30	5.94	10.80	0.0	22.2	47
Planned at the end, 26	2.72	3.50	1.14	0.0	15.40	47
Dif. (initially planned), 26	7.58	6.28	7.60	−5.50	21.10	47
Dentoalveolar expansion upper Arch (mm)						
Initially measured, 16–26	3.36	1.43	3.80	−1.30	6.10	47
Measured after treatment, 16–26	0.34	0.97	0.0	−0.90	3.89	47
Dentoalveolar expansion lower Arch (mm)						
Initially measured, 36–46	2.20	1.97	2.30	−1.10	6.80	47
Measured after treatment, 36–46	−0.08	0.83	0.0	−1.40	3.70	47
Measured in retention, 36–46	−0.06	0.98	−0.10	−1.40	3.70	27
Space recovery upper Arch(mm)						
Initially measured (available space)	2.70	3.26	2.00	0.0	12.0	47
Measured after treatment	0.43	1.00	0.0	0.0	5.50	47
Space recovery lower Arch(mm)						
Initially measured (available space)	1.28	2.83	0.0	0.0	13.0	47
Measured after treatment	0.18	0.59	0.0	0.0	10.0	47
Crowding upper Arch (mm)						
Initially measured (available space)	3.22	3.42	3.00	0.0	14.0	47
Measured after treatment	0.70	1.27	0.0	0.0	5.5	47
Crowding lower Arch (mm)						
Initially measured (available space)	3.12	3.41	1.50	0.0	14.0	47
Measured after treatment	0.54	0.72	0.0	0.0	2.5	47
**Cephalometry (mm)**						
Overbite, initially measured	2.00	3.30	2.70	−7.0	9.0	47
Overbite, measured at the end	3.26	1.14	3.10	0.90	6.30	47
Overjet, initially measured	4.94	2.80	4.80	−1.60	10.70	47
Overjet, measured at the end	3.31	1.14	3.20	1.50	7.90	47
**Skeletal component Cephalometry (°)**						
ANB, initially measured	4.14	2.41	4.60	−1.60	7.90	47
ANB, measured at the end	3.57	2.00	3.80	−2.00	8.20	47
FMA, initially measured	27.64	5.55	27.40	14.60	40.00	47
FMA, measured at the end	26.19	4.87	27.10	13.80	36.5	47
IMPA, initially measured	92.00	8.48	92.50	77.80	114.90	47
IMPA, measured at the end	94.47	6.37	94.20	79.40	107.50	47

Caption: SD: standard deviation; Min: minimum; Max: maximum; *n*: sample size. Planned values correspond to ClinCheck^®^ predicted movements, while measured parameters are the real empirical measurements performed at each time point. To put these measurements in context, the analysis was made in subsets of a sample of 47 children with an average age of 8.6 ± 1.1 years old, 26 females, and 21 males.

**Table 6:** Comparison of cephalometric parameters (ANB, IMPA, FMA, overbite, and overjet) at the initial and end of treatment. (Wilcoxon signed-rank test).

		*n*	Mean Rank	Z	*p*
ANB final (measured) vs. ANB initial (measured)	Negative Ranks	28 ^†^	24.88	−2.021	0.043
	Positive Ranks	17	19.91		
	Ties	2			
	Total	47			
IMPA final (measured) vs. IMPA initial (measured)	Negative Ranks	15 ^†^	19.30	−2.905	0.004
	Positive Ranks	32	26.20		
	Ties	0			
	Total	47			
FMA final (measured) vs. FMA initial (measured)	Negative Ranks	32 ^†^	24.08	−2.513	0.012
	Positive Ranks	14	22.18		
	Ties	1			
	Total	47			
Overbite final (measured) vs. Overbite initial (measured)	Negative Ranks	16 ^†^	18.38	−2.858	0.004
	Positive Ranks	31	26.90		
	Ties	0			
	Total	47			
Overjet final (measured) vs. Overjet initial (measured)	Negative Ranks	36 ^†^	24.29	−3.650	<0.001
	Positive Ranks	10	20.65		
	Ties	1			
	Total	47			

**Caption:** ^†^ Negative ranks represent situations where the final variable measurement is lower than the initial variable measurement; positive ranks represent situations where the final variable measurement is greater than the initial variable measurement; and ties represent situations where the initial values are equal to the final values.

**Table 7:** Comparison of derotation, dentoalveolar expansion, space recovery, and crowding measurements at the initial and end of treatment. (Wilcoxon signed-rank test).

		*n*	Mean Rank	Z	*p*
Derotation molar final (measured) vs. Derotation molar initial (measured), 16	Negative Ranks	44 ^†^	23.80	−5.534	<0.001
	Positive Ranks	2	17.00		
	Ties	1			
	Total	47			
Derotation molar final (measured) vs. Derotation molar initial (measured), 26	Negative Ranks	41 ^†^	19.30	−5.392	<0.001
	Positive Ranks	6	26.20		
	Ties	0			
	Total	47			
Dentoalveolar expansion upper arch final (measured) vs. Dentoalveolar expansion upper arch initial (measured)	Negative Ranks	45 ^†^	24.08	−5.820	<0.001
	Positive Ranks	1	22.18		
	Ties	1			
	Total	47			
Dentoalveolar expansion lower arch final (measured) vs. Dentoalveolar expansion lower arch initial (measured)	Negative Ranks	43 ^†^	18.38	−5.562	<0.001
	Positive Ranks	4	26.90		
	Ties	0			
	Total	47			
Space recovery upper arch final (measured) vs. Space recovery upper arch initial (measured)	Negative Ranks	25 ^†^	24.29	−4.345	<0.001
	Positive Ranks	5	20.65		
	Ties	17			
	Total	47			
Crowding upper arch final (measured) vs. Crowding upper arch initial (measured)	Negative Ranks	11 ^†^		−2.052	<0.001
	Positive Ranks	4			
	Ties	32			
	Total	47			
Crowding lower arch final (measured) vs. Crowding lower arch initial (measured)	Negative Ranks	26 ^†^		−4.434	<0.001
	Positive Ranks	5			
	Ties	16			
	Total	47			

**Caption:** ^†^ Negative ranks represent situations where the final variable measurement is lower than the initial variable measurement; positive ranks represent situations where the final variable measurement is greater than the initial variable measurement; and ties represent situations where the initial values are equal to final values.

## Data Availability

The original contributions presented in this study are included in the article and Supplementary Material. Further inquiries can be directed to the corresponding author.

## Supplementary Materials

The following supporting information can be downloaded at: https://www.mdpi.com/article/10.3390/dj14060338/s1, Table S1: Baseline (T0) and post-treatment (T1) cephalometric measurements, overjet and overbite for the 47 patients included in the cephalometric subsample.

## Abbreviations

The following abbreviations are used in this manuscript:

PAR	Peer Assessment Rating

## References

[1] Thilander B., Lennartsson B. (2002). A Study of Children with Unilateral Posterior Crossbite, Treated and Untreated, in the Deciduous Dentition. J. Orofac. Orthop/Fortschr. Kieferorthop..

[2] Stahl F., Baccetti T., Franchi L., McNamara J.A. (2008). Longitudinal growth changes in untreated subjects with Class II Division 1 malocclusion. Am. J. Orthod. Dentofac. Orthop..

[3] Brierley C.A., DiBiase A., Sandler P.J. (2017). Early Class II treatment. Aust. Dent. J..

[4] Pinho T., Rocha D., Ribeiro S., Monteiro F., Pascoal S., Azevedo R. (2022). Interceptive Treatment with Invisalign^®^ First in Moderate and Severe Cases: A Case Series. Children.

[5] Schneider-Moser U.E.M., Moser L. (2022). Very early orthodontic treatment: When, why and how?. Dent. Press J. Orthod..

[6] Pinho T., Clemente C., Castro I.d., Gonçalves M.d.P. (2025). Retrospective Evaluation of Invisalign^®^ Mandibular Advancement in Growing Patients: Cephalometric, PAR and 3D Molar Displacement Outcomes. Dent. J..

[7] Richmond S., Shaw W.C., Roberts C.T., Andrews M. (1992). The PAR Index (Peer Assessment Rating): Methods to determine outcome of orthodontic treatment in terms of improvement and standards. Eur. J. Orthod..

[8] Papageorgiou S.N., Giannakopoulou T., Eliades T., Vandevska-Radunovic V. (2024). Occlusal outcome of orthodontic treatment: A systematic review with meta-analyses of randomized trials. Eur. J. Orthod..

[9] Kim C.H., Moon S.J., Kang C.M., Song J.S. (2024). The predictability of arch expansion with the Invisalign First system in children with mixed dentition: A retrospective study. J. Clin. Pediatr. Dent..

[10] Inchingolo A.D., Dipalma G., Ferrara I., Viapiano F., Netti A., Ciocia A.M., Mancini A., Malcangi G., Palermo A., Inchingolo A.M. (2024). Clear Aligners in the Growing Patient: A Systematic Review. Children.

[11] McNamara J.A., Franchi L. (2018). The cervical vertebral maturation method: A user’s guide. Angle Orthod..

[12] Weir T. (2017). Clear aligners in orthodontic treatment. Aust. Dent. J..

[13] Dinesh A., Mutalik S., Feldman J., Tadinada A. (2020). Value-addition of lateral cephalometric radiographs in orthodontic diagnosis and treatment planning. Angle Orthod..

[14] Dyken R.A., Sadowsky P.L., Hurst D. (2001). Orthodontic Outcomes Assessment Using the Peer Assessment Rating Index. Angle Orthod..

[15] Lanteri V., Farronato G., Lanteri C., Caravita R., Cossellu G. (2018). The efficacy of orthodontic treatments for anterior crowding with Invisalign compared with fixed appliances using the Peer Assessment Rating Index. Quintessence Int..

[16] Freitas K.M.S., Freitas D.S., Valarelli F.P., Freitas M.R., Janson G. (2008). PAR evaluation of treated class I extraction patients. Angle Orthod..

[17] da Silva V.M., Ayub P.V., Massaro C., Janson G., Garib D. (2023). Comparison between clear aligners and 2 × 4 mechanics in the mixed dentition: A randomized clinical trial. Angle Orthod..

[18] Loberto S., Pavoni C., Fanelli S., Lugli L., Cozza P., Lione R. (2024). Predictability of expansion movements performed by clear aligners in mixed dentition in both arches: A retrospective study on digital casts. BMC Oral Health.

[19] Levrini L., Zecca P.A., Borgese M., Scurati E.I., Deppieri A., Saran S., Caccia M., Carganico A. (2025). Efficacy of Maxillary Expansion with Clear Aligner in the Mixed Dentition: A Systematic Review. Appl. Sci..

[20] Torbaty P.M., Suh H., Tai S.K., Baird M., Boyd R.L., Oh H. (2024). Vertical and transverse treatment effects of Invisalign First system compared to Hyrax maxillary expanders with fixed appliances in mixed dentition patients. Angle Orthod..

[21] Pamukçu H., Özsoy S., Aksoy P.C., Özsoy Ö.P. (2024). Evaluation of maxillary dimensional changes in the mixed dentition: Clear aligners vs acrylic expanders. Angle Orthod..

[22] Mirzen Arat Z., Rü Bendü Z.M. (2005). Changes in Dentoalveolar and Facial Heights during Early and Late Growth Periods: A Longitudinal Study. Angle Orthod..

[23] Levrini L., Carganico A., Abbate L. (2021). Maxillary expansion with clear aligners in the mixed dentition: A preliminary study with Invisalign^®^ First system. Eur. J. Paediatr. Dent..

[24] D’Antò V., De Simone V., Caruso S., Bucci P., Valletta R., Rongo R., Bucci R. (2024). Effects of clear aligners treatment in growing patients: A systematic review. Front. Oral Health.

[25] Bruni A., Ferrillo M., Gallo V., Parrini S., Garino F., Castroflorio T., Deregibus A. (2024). Efficacy of clear aligners vs rapid palatal expanders on palatal volume and surface area in mixed dentition patients: A randomized controlled trial. Am. J. Orthod. Dentofac. Orthop..

[26] Graf I., Puppe C., Schwarze J., Höfer K., Christ H., Braumann B. (2021). Evaluation of effectiveness and stability of aligner treatments using the Peer Assessment Rating Index. J. Orofac. Orthop..

