# Supplementary material for: Longitudinal Changes in Occlusal Outcomes and Case Complexity with Invisalign® First in Early Mixed Dentition
Source: Dent J (Basel). 2026 Jun 2;14(6):338. doi: 10.3390/dj14060338 (PMC13298412; doi:10.3390/dj14060338)
Supplement: Supplementary file 1 [file dentistry-14-00338-s001.zip › dentistry-4265406-supplementary/Table S1 Supplementary Material.pdf]

Supplementary Tables

Supplementary Table S1. Baseline (T0) and post-treatment (T1) cephalometric measurements, overjet and overbite for the 47 patients included in the cephalometric subsample.

Cases	ANB T0 3º ± 2	ANB T1 3º ± 2	IMPA T0 90º ± 5º	IMPA T1 90º ± 5º	FMA T0 25º ± 3º	FMA T1 25º ± 3º	Overbite T0 2,5 + 2,5	Overbite T1 2,5 ± 2,5	Overjet T0 2,5 + 2,5	Overjet T1 2,5 ± 2,5
Case 1	2.1	4	82.2	90.6	32.5	28.3	-3.6	3	0.7	3.7
Case 2	6.6	3.4	87.3	94.1	40	28.8	-4.1	1.9	6	3.3
Case 3	7.8	3.1	80.2	89.7	24.7	27.1	8.7	3.6	5.3	4
Case 4	3.5	0.6	81.8	78.4	30.7	29.4	4.7	2.9	4.2	2.7
Case 5	-1.5	(-2.9)	78.8	87.4	20	13.8	2.1	3.8	3.2	3.5
Case 6	4.8	5	92.5	92.2	30.9	27.7	3.8	4.1	9.4	4.3
Case 7	3.7	3.8	101.6	102.2	16.6	15.2	6.2	6.3	10.3	7.9
Case 8	6	4.6	97	98.3	27.7	27.4	-0.7	2.1	10.3	3.4
Case 9	6.3	4.8	99.2	100.7	32.4	27.3	-7	1.3	1.5	2.4
Case 10	5.2	5.2	104.4	100.5	20.9	23.6	3.4	4.2	4.9	4.9
Case 11	3	3.4	97	91.3	26	31.8	2	4	6.5	3.2
Case 12	0.9	2.8	89.6	85.2	27.6	27.6	1.7	2.7	-1.6	4.3
Case 13	3.3	1	78	86	26	24.7	3.6	3.8	3.5	3.2
Case 14	3.8	4.9	87.9	95.6	32.6	30.7	4.3	4.7	7.6	3.6
Case 15	4.6	3.3	93.2	96.9	26.4	27	5.6	5.5	4.6	4.5
Case 16	-1.6	0.4	84.2	88.5	33.9	30.9	0	2.7	0.3	3.4
Case 17	6.4	3.2	101.3	96.2	36	36.5	0.9	2.3	6	3.2
Case 18	6.8	3.1	98.3	98.8	23.9	21.6	0.6	3.9	6.9	4.3
Case 19	-0.3	(-0.4)	87.3	89.2	27.4	29.5	1.3	3.5	1.3	4.1
Case 20	5.9	4.6	97.7	101.9	30.9	31.1	5.1	3.7	5.9	3.6
Case 21	7	6	99.8	104.4	24.1	22.1	2.1	4.3	5.9	2.9
Case 22	4.4	4	114.9	107.5	14.6	15.5	4.2	3.4	3.3	2.6
Case 23	6	4.4	100.4	96.1	24.9	26.6	5.5	5.2	4.2	4.4
Case 24	2.2	1.9	92	92.6	23.2	24.7	0	2.6	0	2.5
Case 25	-0.1	1.1	91.3	90.1	24.7	27.1	0.3	2.8	3.2	3.1
Case 26	5.4	3.9	83	88.7	34.9	28	-3.3	2.3	10.7	2.8
Case 27	2	1.7	83.1	87.3	24.5	24.2	3.9	2.5	4.1	2
Case 28	7.9	6.2	98.8	98.4	30.3	28.3	3.6	4.1	8.8	4.1
Case 29	4.1	4.6	87.3	91.1	24.7	23.1	6.5	3.4	4.8	2.6
Case 30	3.2	4.3	83.3	88.3	26.6	24.1	4	3	3.8	2.3
Case 31	2.6	1.5	86.5	90.7	30	27.8	4.5	3.1	7.2	3.1
Case 32	2.8	1.5	86.1	87.2	29.1	26.8	0.1	2.7	1.7	3.0
Case 33	7.4	4.6	84.2	94.2	31.9	34.5	-2.9	1.5	8.7	2.5
Case 34	1.2	2.1	81.2	89.5	24	22.7	4.3	2.3	3.5	2
Case 35	4.7	4.7	85.6	95.4	28.4	31.3	2.7	1.9	4.2	2.6
Case 36	3.6	6.4	86.5	92.5	39.6	33.3	-0.9	2.6	5.5	1.5
Case 37	4.6	5	94.3	92	38.2	33.2	2	3.8	4.3	4.0
Case 38	7.2	8.2	92.8	95.5	29.2	28	-2.4	3.4	5	3
Case 39	7	6	98.5	104.5	21.2	20.3	3.9	2.5	3.8	2.3
Case 40	5.1	3.7	100.3	99.3	19.7	19.4	4.9	3.2	3.4	2.5
Case 41	5.2	7.3	100.3	101.6	27.1	25.6	-5	0.9	6.2	1.7
Case 42	1.4	4.7	96.1	106.5	28.3	22	4	4.5	5.3	3.4
Case 43	3.5	3.2	104.4	102.3	22.9	27.9	4	3.0	3.4	3.2
Case 44	7.3	4.9	99.5	100.7	24.4	23.6	4.3	4	5.9	3.7
Case 45	6.2	2.5	97.5	97.2	33	22.7	3.2	5.7	9.4	3.3
Case 46	1.9	1.7	77.8	86	30.3	28	1.7	2.4	2.2	1.7
Case 47	5.3	2.9	98,8	96.7	22	19.4	0.1	2.0	6.7	2.2
